# Mosquito-borne heartworm *Dirofilaria immitis* in dogs from Australia

**DOI:** 10.1186/s13071-016-1821-x

**Published:** 2016-10-07

**Authors:** Chloe Nguyen, Wei Ling Koh, Andrea Casteriano, Niek Beijerink, Christopher Godfrey, Graeme Brown, David Emery, Jan Šlapeta

**Affiliations:** 1 Faculty of Veterinary Science, The University of Sydney, McMaster Building B14, Sydney, 2006 New South Wales Australia; 2 University Veterinary Teaching Hospital, Sydney (UVTHS), Faculty of Veterinary Science, The University of Sydney, Evelyn Williams Building B10, Sydney, 2006 New South Wales Australia; 3 School of Life and Environmental Sciences, Faculty of Veterinary Science, The University of Sydney, McMaster Building B14, Sydney, 2006 New South Wales Australia

**Keywords:** *Dirofilaria immitis*, *Acanthocheilonema reconditum*, Drug resistance, PCR, Knott’s test, High resolution melt qPCR

## Abstract

**Background:**

Heartworm (*Dirofilaria immitis*) in dogs is considered endemic in Australia, but the clinical heartworm disease caused by the heartworm is rare and prevalence is low. The mainstream prevention of the heartworm is based on macrocyclic lactone (ML) administration. The aim of this study was to confirm endemism of the heartworm under current Australian conditions using a cohort of recent microfilaria-positive dogs which were on variable heartworm prevention.

**Methods:**

A hotspot of canine heartworm antigen-positive and microfilaria-positive dogs has been detected recently in Queensland, Australia. Blood samples from 39 dogs from Queensland and two dogs from New South Wales were investigated for canine filarioids. Rapid antigen diagnostic tests capable of detection of *D. immitis* and real-time PCR for quantification and differentiation between *D. immitis* from *Acanthocheilonema reconditum* with quantification of microfilariae in canine blood samples, together with *D. immitis* specific real-time PCR assay, were applied to microfilaria-positive dogs. The P-glycoprotein genotype was determined to test whether Australian-sourced heartworm shared the same genetic markers as those suspected of ML-resistance in North America.

**Results:**

Only *D. immitis* was detected in the samples from Queensland and New South Wales, Australia. Using high resolution melt real-time PCR and *D. immitis* specific real-time PCR, the calculated microfilaria concentration ranged from 1 to 44,957 microfilariae/ml and from 7 to 60,526 microfilariae/ml, respectively. DNA sequencing of the PCR products confirmed *D. immitis.* Fifteen of the examined dogs were on putative, rigorous ML prevention. For the remaining dogs, compliance with heartworm prevention was unknown or reported as inconsistent. Wild-type genotype AA-GG of the P-glycoprotein locus of *D. immitis* sequence has been obtained for three blood samples. Due to the incomplete history, any suggestion of a loss of efficacy of MLs must be treated as ‘remotely possible’. In the immediate future, records of preventative administration and annual antigen testing would be required to determine any problems with the efficacy of preventatives.

**Conclusions:**

The prevalence of canine heartworm in Australia remains poorly understood. It is generally assumed to be low by veterinary practitioners. The localised increase in the study area confirms endemism of canine heartworm and a requirement for ongoing vigilance through annual heartworm testing to better understand the changing distribution of canine heartworm, client compliance, as well as to detect any change in ML-susceptibility.

**Electronic supplementary material:**

The online version of this article (doi:10.1186/s13071-016-1821-x) contains supplementary material, which is available to authorized users.

## Background

*Dirofilaria immitis* (canine heartworm) is a parasitic nematode responsible for the canine heartworm disease. Transmitted from host-to-host *via* mosquito bites, infection largely affects the cardiopulmonary system resulting in severe and potentially fatal disease. The disease is well-established, mostly affecting temperate, tropical and subtropical areas of North, Central and South America, southern Europe, as well as Australia [1–7]. The prevalence of the disease is influenced by climate and topography, but most importantly, by the presence of mosquito vectors - ubiquitous *Culex* spp., *Aedes* spp. and *Anopheles* spp. [6, 8]. Transmission occurs when mosquitoes ingest *D. immitis* microfilariae (L1) as they circulate the blood of infected animals. In the mosquito, the microfilariae moult before migrating to the proboscis as *D. immitis* infective third-stage larvae (L3) [9]. These larvae (L3) are transferred to hosts during blood meals, where migrate throughout the blood and body towards the pulmonary arteries and reach sexual maturity in large blood vessels [10]. The microfilariae are first seen in 6–7 month after the initial infection and the life span of adult female (230–310 mm in length) and male (120–190 mm in length) heartworms is 5–10 years [8–10].

The principal prevention for heartworm in dogs is based on monthly oral or topical administration of macrocyclic lactones (ML) or yearly injections of moxidectin microspheres (also ML). Canine heartworm is considered endemic in Australia, with the principal mosquito vectors being *Aedes notoscriptus* and *Culex annilirostris* in southeast Australia [11]. Between 1970 and 1990, there was a reported increase in heartworm prevalence from 5 % to a peak of 30 % in New South Wales [12–14]. Heartworm was consistently found across all states and territories with highest rates of prevalence found in Queensland (36 %) and Northern Territory (90–100 %) [1, 15–18]. Current prevalence is low even in sentinel populations such as Aboriginal community dogs [19]. Australian dingos/wild dogs potentially represent a reservoir in northern Queensland based on recent small scale survey [20]. A recent 2011 heartworm antigen survey found only 2 (0.3 %) heartworm-positive dogs out of 783 pound dogs from New South Wales, Queensland and South Australia with no known history of prophylaxis (Sarah Cooper, Australian Veterinary Association Annual Conference, 20–25 May 2012, Canberra). Based on the above surveys, Australia should be considered a region of low prevalence for heartworm in dogs.

Since 2005, a lack of efficacy of MLs to prevent heartworm infection has been reported in the USA, suggesting the possibility that heartworm is developing resistance to ML-preventatives [21]. Studies since have been focused on identification of potential molecular changes that could be associated with ML lack of efficacy [22–25]. Comparisons of ML-resistant and susceptible *D. immitis* have identified possible single nucleotide polymorphisms (SNPs) such as the GG-GG genotype in the P-glycoprotein (P-gp) with ivermectin-resistance [22, 23, 26]. More recently, whole genome scans discovered over 150 markers potentially associated with ML-resistance [27]. However, further research is required to validate these genetic markers and their utility for diagnostic screening [7]. At present, no genetic test is available to differentiate ML-susceptible from ML-resistant field specimens. Compared to USA, lack of efficacy of MLs to prevent canine heartworm has not been reported in Australia.

The aim of this study was to confirm endemicity of the canine heartworm under current Australian conditions using a cohort of recent heartworm-positive dog material which were on variable heartworm prevention. Rapid diagnostic tests capable of detection, differentiation and quantification of microfilaria and species specific real-time PCR assays were applied to blood or microfilaria from positive dogs. The P-gp genotype was determined to ascertain whether these Australian heartworms shared the same genotype as suspected ML-resistant heartworm in North America.

## Methods

### Samples

Samples tested included two cohorts. The first cohort, samples (FVS-CN, *n* = 39) were from those that tested positive using in clinic antigen tests. The second cohort, samples (FVS-HW, *n* = 2) were from recent clinical cases currently treated for canine heartworm at the Faculty of Veterinary Science, University of Sydney.

Blood samples FVS-CN#1 to FVS-CN#39 were collected in April to June, 2015, from dogs in Queensland, Australia (Table 1). Dogs were located in the Mackay region (FVS-CN#1, FVS-CN#3 to FVS-CN#34, FVS-CN#36 to FVS-CN#39), Cairns (FVS-CN#2) and Toowoomba (FVS-CN#35) (Additional file 1: Table S1). All samples initially tested positive using an in-clinic Witness Dirofilaria® (Zoetis, Australia), a Rapid Immuno-Migration (RIM®) Assay for detection of adult female *D. immitis* (heartworm) antigen in dogs positive by veterinary practitioners. To confirm the result, we performed DiroCHEK® (Zoetis, Australia via Vetpath Laboratory Services, Western Australia) an enzyme-linked immunosorbent assay (ELISA) for the detection of adult *D. immitis* antigen and Knott’s test (Vetpath Laboratory Services, Western Australia). For each dog only locality, the age, presence of suspected heartworm associated clinical signs and history of heartworm prevention were available. Heartworm prevention compliance was reported as ‘rigorous’ if the client indicated year round administration with appropriate product (no actual evidence of purchase and administration was provided), ‘inconsistent’ if heartworm preventions were missed or intermittently administrated or incorrect dose administered, and ‘unknown’ if such information was not available or was unknown. Anonymous refrigerated blood samples FVS-CN#1 to FVS-CN#39 were processed at the Faculty of Veterinary Sciences, University of Sydney for this study (Additional file 1: Table S1).

**Table 1:** Summary of diagnostics for heartworm (*Dirofilaria immitis*) in dogs from Australia

Animal (dogs)		Immunology		Parasitology	Molecular diagnostics (real-time PCR)				Heartworm prevention	
FVS-CN#	Age (yrs)	Witness^a^	DiroCHEK^b^	Knott’s test	Dog (C_t_)^c^	Filaria (Mff#/ml)^d^	*D. immitis* (Mff#/ml)^e^	Clinical signs	Compliance^f^	Active
1	6.0	Positive	Positive	Positive	–	–	7	–	Inconsistent	na
2	9.0	Positive	Positive	Positive	–	–	–	Present	Yes	Selamectin
3	2.0	Positive	Positive	Positive	27.6	10	14	–	na	na
4	7.5	Positive	Positive	Positive	35.0	165	147	Present	na	na
5	3.0	Positive	Positive	Positive	31.1	10	9	–	na	na
6	6.7	Positive	Positive	Positive	26.8	–	–	–	Yes	Milbemycin oxime
7	8.0	Positive	Positive	Positive	26.4	835	571	–	Yes	Ivermectin
8	10.0	Positive	Positive	Positive	24.9	215	204	–	na	na
9	3.8	Positive	Positive	Positive	26.2	118	52	–	Yes	Milbemycin oxime
10	5.1	Positive	Positive	Positive	27.7	46	15	–	inconsistent	na
11	na	Positive	Positive	Positive	24.5	23,376	60,527	–	inconsistent	na
12	2.8	Positive	Positive	Positive	30.2	24	460	–	Yes	Moxidectin
13	2.0	Positive	Negative	Positive	24.3	126	77	Present	inconsistent	na
14	4.0	Positive	Positive	Positive	32.9	3	0	–	na	na
15	7.0	Positive	Positive	Positive	22.4	912	610	–	na	na
16	2.0	Positive	Positive	Positive	23.0	940	261	–	na	na
17	3.0	Positive	Positive	Positive	24.9	165	518	–	na	na
18	2.0	Positive	Negative	Positive	25.2	124	239	–	na	na
19	2.0	Positive	Positive	Positive	24.6	34	261	Present	Yes	Milbemycin oxime
20	3.0	Positive	Positive	Positive	26.3	1,184	3,471	–	na	na
21	2.0	Positive	Positive	Positive	28.6	36	130	–	Yes	Milbemycin oxime
22	13.0	Positive	Positive	Positive	28.2	70	52	Present	Yes	Milbemycin oxime
23	2.0	Positive	Positive	Positive	28.8	60	416	–	Yes	Milbemycin oxime
24	3.0	Positive	Positive	Positive	38.8	1	77	–	na	na
25	5.0	Positive	Positive	Positive	26.6	4,543	8,042	Present	inconsistent	na
26	3.0	Positive	Positive	Positive	24.5	58	319	–	inconsistent	na
27	3.0	Positive	Positive	Positive	34.1	16	157	–	inconsistent	na
28	10.0	Positive	Positive	Positive	28.8	11,214	1,478	–	inconsistent	na
29	2.0	Positive	Positive	Positive	36.7	–	12	–	na	na
30	10.0	Positive	Positive	Positive	24.3	2,022	9,551	–	Yes	Ivermectin
31	7.0	Positive	Positive	Positive	23.9	177	217	–	na	na
32	6.1	Positive	Positive	Positive	24.1	15	321	–	Yes	Milbemycin oxime
33	na	Positive	Positive	Positive	28.8	41	45	Present	inconsistent	Ivermectin
34	5.5	Positive	Positive	Positive	23.7	8,745	15,976	Present	Yes	Milbemycin oxime
35	6.0	Positive	Positive	Positive	37.4	198	320	–	n/a	na
36	4.0	Positive	Positive	Positive	24.8	44,966	1,866	–	n/a	na
37	2.1	Positive	Positive	Positive	35.6	31	5,717	–	Yes	Milbemycin oxime
38	7.0	Positive	Positive	Positive	28.8	142	4,505	–	Yes	Moxidectin
39	1.8	Positive	Positive	Positive	27.4	773	276	–	Yes	Milbemycin oxime

^a^In-clinic Witness Dirofilaria® (Zoetis, Australia), a Rapid Immuno-Migration (RIM®) Assay for detection of adult female *D. immitis* (heartworm) antigen in dogs

^b^DiroCHEK® (Zoetis, Australia) an enzyme-linked immunosorbent assay (ELISA) for the detection of adult *D. immitis* antigen

^c^Canine ß-actin real-time PCR assay for sample adequacy evaluation, C_t_ <30 is considered adequate (*n* = 28, 72 %)

^d^Arthropod-borne filarioids high-resolution melt (HRM) real-time PCR assay targeting filarioid mitochondral 12S rRNA gene, reported as microfilaria (Mff) in ml of blood

^e^ *D. immitis* species-specific real-time PCR targeting *cox*1 gene fragment, reported as microfilaria (Mff) in ml of blood

^f^Rigorous compliance (Yes) was noted if client indicated year round administration with appropriate product, ‘inconsistent’ compliance if heartworm preventions were missed or intermittently administrated or incorrect dose administered, and ‘na’ if such information was not available or was unknown

Two recent (February, 2016) microfilaria-positive dog blood samples were provided by the University Veterinary Teaching Hospital, Sydney (UVTHS), Faculty of Veterinary Science, University of Sydney. Both dogs were previously domiciled in Queensland, Australia. For both FVS-HW#1 (1,300 microfilariae/ml) and FVS-HW#2 (14 microfilariae/ml) the microfilaria concentration was evaluated using microcapillary test. Briefly, the buffy coat was observed for the presence of microfilaria under the microscope in microhematocrit tubes after centrifugation of EDTA blood. FVS-HW#1 positivity was also assessed with modified Knott’s test. Dog FVS-HW#1 was referred to UVTHS for heartworm disease prior to treatment, blood was collected and tested positive using Anigen Rapid Canine Heartworm (CHW) Ag 2.0 Test Kit (Bionote; Life Bioscience, Oakleigh, Australia) a chromatographic immunoassay for the qualitative detection of canine *D. immitis* antigen; the dog stayed in Mackay, Queensland prior to arriving in Sydney, New South Wales. Dog FVS-HW#2 was undergoing heartworm treatment with melarsomine (Immiticide®, Merial), and blood was collected prior to second treatment, at the time blood of FVS-HW#2 was negative using Anigen Rapid Canine Heartworm (CHW) Ag 2.0 Test Kit; the dog was from Brisbane, Queensland but it was rehomed from elsewhere (unknown source) in Queensland. For both FVS-HW#1 and FVS-HW#2, heartworm prevention was intermittent. In addition, canine DNA isolated from a dog domiciled in Nadi, Fiji (FIJI-HW#1) from 2009 was included in the study as initial positive control.

### Isolation and confirmation of canine blood DNA

Blood samples (EDTA, 70–200 μl) from FVS-CN#1 to FVS-CN#39 were kept frozen for 8–10 months at -20 °C and refrigerated blood (200 μl) from FVS-HW#1 and FVS-HW#2 was used for DNA isolation. The genomic DNA from samples FVS-CN#7 to FVS-CN#39, FVS-HW#1 and FVS-HW#2 was isolated using a combination of MoBio PowerMag® Blood Isolation Kit (lysis) and PowerMag® Microbiome RNA/DNA Isolation Kit (magnetic separation) (Mo-Bio Laboratories; GeneWorks, Thebarton, Australia) optimised for KingFisher® Duo (Thermo-Fisher Scientific, Scoresby, Australia). Isolation was processed in batches of 11 blood samples and an extraction blank. Initial lysis was according to the Blood kit isolation part with modification to the step of white blood cell (WBC) lysis (650 μl of the red blood cell lysate and 450 μl of PowerMag® WBC lysis solution). DNA isolation was processed with 700 μl of the WBC supernatant in the KingFisher® Microtiter Deep Well 96 plate as suggested by the Microbiome kit protocol. Samples were eluted in 70 μl of ClearMag® RNase-Free water. Genomic DNA from samples FVS-CN#1 to FVS-CN#6 was isolated using Isolate II Blood DNA Kit (BioLine, Australia) and eluted in 100 μl Tris buffer (pH = 8) as per the manufacturer’s protocol. DNA concentrations were determined by the NanoDrop 1000 Spectrophotometer (Thermo-Fisher Scientific, Scoresby, Australia). DNA was stored in aliquots at -20 °C until further use.

Buffy coat with 1–2 microfilaria from FVS-HW#2 was collected using a tuberculin syringe and needle from the micro-haematocrit tube after centrifugation. DNA was isolated using the MoBio Kits on KingFisher as described above.

To verify presence of dog DNA, we utilised a real-time PCR to amplify partial (276 bp) canine ß-actin, primers (S0588) Actin F2: 5′-ACC ACT GGT ATT GTC ATG GAC TCT G-3′ and (S0589) Actin R2: 5′-GCT CTT CTC CAG GGA GGA CGA-3′ [28]. The PCR cycling conditions included initial step at 95 °C for 3 min followed by 40 two-step cycles at 95 °C for 5 s and 60 °C for 15 s. The real-time PCR reaction mixture (20 μl) included 10 μl of SensiFAST SYBR No-ROX Kit (Bioline, Eveleigh, Australia), each primer at 400 nM concentrations, PCR-grade water, and 2 μl of DNA template. The real-time PCR was carried out on a CFX96 Touch™ Real-Time PCR Detection System with the corresponding CFX Manager v.3.1 software (BioRad, Gladesville, Australia). The arbitrary real-time PCR threshold was determined automatically using default settings and C_t_-values reported.

### Arthropod-borne filarioids real-time PCR

A high-resolution melt (HRM), real-time PCR assay was performed using primers designed by Wongkamchai et al. [29] for the detection of *Dirofilaria immitis*, *Brugia malayi* and *B. pahangi* in blood samples. Primers (S0579) (F: 5′-TTT AAA CCG AAA AAA TAT TGA CTG AC-3′) and (S0580) (R: 5′-AAA AAC TAA ACA ATC ATA CAT GTG CC-3′) target approximately 110–120 bp long partial sequence of the filarioid mitochondrial 12S rRNA gene. The assay is capable of amplifying potentially all filarial nematode species [29, 30]. The real-time PCR reaction mixture (20 μl) included 10 μl of SensiFAST HRM mix (Bioline), each primer at 400 nM concentrations, 4.4 μl of PCR-grade water, and 4 μl of DNA template. Positive control plasmids with DNA for *D. immitis* and *Acanthocheilonema reconditum* 12S rRNA gene were synthetised by GeneArt (Thermo-Fisher Scientific, Scoresby, Australia).

To optimize conditions with SensiFAST HRM mix (BioLine, Australia), real-time PCR efficiency was evaluated across thermal gradient from 59 °C to 62 °C against ten-fold serial dilutions (ranging from 1.96 × 10^5^ to 1.96 × 10^0^ copies per reaction) of a known concentration of plasmid DNA containing the *D. immitis* 12S rRNA gene analysed in triplicate. The reaction conditions were set with an initial polymerase activation step at 95 °C for 3 min followed by 40 two-step cycles at 95 °C for 5 s and 59 °C to 62 °C for 15 s. The HRM cycle was set between 60 °C and 90 °C, with increments at 0.2 °C intervals. The real-time PCR was carried out on a CFX96 Touch™ Real-Time PCR Detection System with the corresponding CFX Manager v. 3.1 software (BioRad, Australia). For each sample CFX Manager v. 3.1 reported C_t_-values and copy numbers based on standard curve of the positive control. The arbitrary real-time PCR threshold was set to a single threshold at 100 rfu. To plot a normalised curve of decreasing fluorescence with increasing temperature the Precision Melt Software v. 1.2 (Bio-Rad, Australia) was used. Each run included at least one negative (NTC, non-target control) control.

The optimised filarial 12S rRNA gene real time PCR was run as above with annealing temperature set to 62 °C with reaction efficiency (90–110 %) and the limit of detection determined to be greater or equals to 196 gene copies of 12S rRNA gene per reaction. The optimised real-time PCR was used to quantify microfilaria concentrations in blood samples and sample FVS-HW#2 (1,300 microfilariae/ml) used to calibrate conversion to microfilaria for each sample (Additional file 1: Table S1). Each real-time PCR run included duplicate serial dilutions of plasmid DNA (*D. immitis*, *A. reconditum* plasmids). All DNA samples were run once. If no or > 35 C_t_-values (negative samples and late amplifiers, respectively) were returned, the reactions were re-run. All samples with < 35 C_t_-values were sent for DNA purification and subsequent DNA sequencing with amplification primers at Macrogen Inc. (Seoul, Korea). To generate normalised melting curves and difference melting curves, pre- (70–71 °C) and post- (76–77 °C) melt normalisation regions were set to define the main temperature boundaries of the normalised and difference plots. The Precision Melt Software v. 1.2 then assigned each sample to a cluster using default settings.

### *Dirofilaria immitis* specific real-time PCR

A real-time PCR assay was performed for detection of *D. immitis* using primers designed by Rishniw et al. [28] and applied by Rojas et al. [30]. Primers (S0582) (F: 5′-AGT GTA GAG GGT CAG CCT GAG TTA-3′) and (S0583) (R: 5′-ACA GGC ACT GAC AAT ACC AAT-3′) specifically target a *cox*1 gene fragment (~203 bp) for *D. immitis*. The real-time PCR reaction mixture (20 μl) included 10 μl of SensiFAST SYBR No-ROX Kit (Bioline, Australia), each primer at 400 nM concentrations, 4.4 μl of PCR-grade water, and 4 μl of DNA template. First, a thermal gradient PCR assay from 59 °C to 62 °C was performed to determine optimum annealing temperatures for the pair of primers using SensiFAST SYBR No-ROX kit (Bioline, Australia). The reaction was run with triplicate ten-fold serial dilutions (1.96 × 10^2^ to 1.96 × 10^7^ copies per reaction) of a known concentration of plasmid DNA containing the *D. immitis cox*1 gene synthetised by GeneArt (Thermo Fisher, Australia) and with negative (no template) controls. Amplification was performed as follows: initial denaturing step at 95 °C for 3 min, followed by 40 cycles of denaturing (5 s at 95 °C), annealing (15 s from 59 °C to 62 °C). A final melting curve was produced by heating the product from 60 °C to 90 °C for 5 s at 0.5 °C increments. The real-time PCR assay was performed on a CFX95 Touch™ Real-Time PCR Detection System (BioRad Laboratories, Inc., Australia). Threshold cycle (C_t_) values and standard curve were determined CFX Manager ™ Software v. 3.1 (Bio-Rad). An annealing temperature of 62 °C was found optimal and used in subsequent amplification reactions.

The final optimised real-time PCR run consisted of samples FVS-CN#1 to FVS-CN#39, a no-template control and a non-target control with additional duplicate ten-fold serial dilutions of the plasmid DNA (1.96 × 10^7^ to 1.96 × 10^2^ copies per reaction) to assess sensitivity. The optimised real time PCR included annealing temperature set to 62 °C with reaction efficiency (90–110 %) and the limit of detection determined to be ≥ 196 *cox*1 gene copies per reaction. Using CFX Manager ™ Software Version 3.1 (Bio-Rad), C_t_-values were determined and a standard curve produced to evaluate assay efficiency. Samples which amplified with C_t_-values were sent for DNA purification and sequencing at Macrogen Inc. (Seoul, Korea).

### Characterisation of *Dirofilaria immitis* P-glycoprotein locus sequence

To obtain PCR amplicons of P-glycoprotein locus of *D. immitis* (P-gp) we initially used DNA directly isolated from blood PCR. To improve success of the P-gp amplification, we then used a whole genome amplification step of the DNA isolated from blood. Whole genome amplification (WGA) was carried out using the illustra GenomiPhi V2 DNA Amplification Kit (GE Healthcare, Parramatta, Australia) using 1 μl of gDNA extracted from the canine blood. For WGA, we primarily focused on DNA samples with reported rigorous compliance with heartworm preventatives (Table 1). Amplification of the P-pg fragment was accomplished with primer pair, Pgp1sens (S0592) (5′-GGA CAA TTA TCC GGT GGT CA-3′) and Pgp1antisens (S0593) (5′-TCG CAA ATT TCC TCC CAC TT-3′) [22, 23]. All PCR amplifications were carried out using MyTaq™ Red Mix (BioLine, Australia). Primers were added at a final concentration of 0.25 μM each. A final reaction volume of 25 μl for the first PCR was run using the following cycling conditions: 95 °C for 15 s, 56 °C for 5 s, and 72 °C for 15 s for 35 cycles with 2 μl of the isolated DNA or 2 μl of 1:50 dilution of the GenomiPhi amplified DNA. All reactions were initiated at 95 °C for 5 min and concluded with 72 °C for 5 min. The PCRs were run in a Veriti PCR cycler (Life Sciences, Australia) alongside sterile PCR-grade water as negative control. Resulting products were resolved in 1.5 % (w/v) agarose gel. The PCRs were run in a Veriti PCR cycler (Life Sciences, Australia) alongside sterile PCR-grade water as negative control. Resulting products were resolved in 1.5 % (w/v) agarose gel. PCR products were directly sequenced using amplification primers at Macrogen Ltd. (Seoul, Korea). Sequences were assembled, aligned with a reference wild type P-gp locus sequence (HM596853) and/or BLAST analysed using CLC Main Workbench 6.9.1 (CLC bio, Denmark).

## Results

### Patent microfilaraemic dogs in coastal Queensland, Australia

**Fig. 1:**
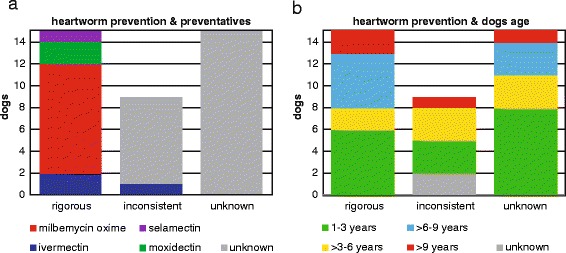
Canine heartworm *Dirofilaria immitis* in Australian dogs. Heartworm prevention compliance for 39 dogs was reported as ‘rigorous’ compliance if client indicated year round administration with appropriate product (no actual evidence of purchase and administration was provided), ‘inconsistent’ compliance if heartworm preventions were missed or intermittently administrated or incorrect monthly dose administered, and ‘unknown’ if such information was not available or was unknown. The active in prevention of dog heartworm is colour indicated (**a**). The age of dogs is colour indicated (**b**)

All 39 dogs (FVS-CN#1 to FVS-CN#39) were initially positive using an in-clinic Witness Dirofilaria® (Zoetis, Australia) test for presence of female *D. immitis* antigen. A reference ELISA DiroCHEK® (Zoetis, Australia) confirmed presence of female *D. immitis* antigen in 95 % (37/39, 2 false negative samples: FVS-CN#13 and FVS-CN#18) of dogs and microfilaria were demonstrated in all 39 samples using a modified Knott’s test (Table 1, Fig. 1).

In the dataset presented with microfilaria (FVS-CN#1 to FVS-CN#39), 41 % of dogs were apparently compliant with monthly heartworm prevention (*n* = 15) (Fig. 1a). For the remaining dogs compliance with heartworm prevention was unknown or reported as inconsistent (Table 1). Most heartworm prevention was based on milbemycin oxime (67 %, *n* = 10) followed by ivermectin (*n* = 2), moxidectin (*n* = 2) and selamectin (*n* = 1). The average age of the dogs was 5 years 6 month (min. 1 year 9 month; max. 13 years); seventeen dogs (43 %) were three years or less (five on monthly milbemycin oxime, one on monthly moxidectin) (Fig. 1b). Eight dogs were in the category of over six years and up to nine years (two on monthly milbemycin oxime, and single dogs on moxidectin, selamectin and ivermectin) (Additional file 1: Table S1).

DNA from FVS-CN#1 to FVS-CN#39 was successfully purified and canine ß-actin amplified (C_t_ values: 22.44 to 28.84) from 72 % (28/39) canine blood samples. Nine (23 %, 9/39) DNA from blood samples amplified DNA with higher C_t_-value (< 30), and two (5 %, 2/29) DNA samples (FVS-CN#1 and FVS-CN#2) did not amplify canine ß-actin control gene (Table 1). FVS-HW#1, FVS-HW#2 and FIJI-HW#2 returned adequate C_t_-values 25.88, 29.76 and 25.31 in canine ß-actin assay, respectively. All blank DNA samples remained negative in the canine ß-actin assay.

### Presence of *Dirofilaria immitis* using arthropod-borne filarioids real-time PCR with HRM and DNA sequencing

**Fig. 2:**
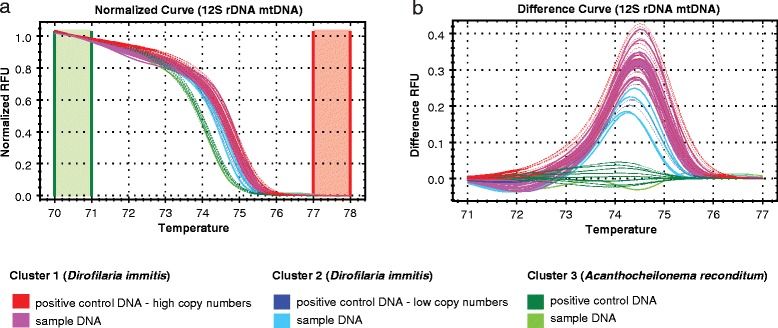
HRM real-time qPCR analysis for the identification of arthropod-borne filarioids in Australia dogs. Normalized melting curves (**a**) and difference melting curves (**b**). Curves include positive controls of *Acanthocheilonema reconditum* (*green*) and *Dirofilaria immitis* (*red*, *blue*). To generate normalised melting curves and difference melting curves, pre- (70–71 °C) and post-melt (76–77 °C) normalisation regions were set to define the main temperature boundaries of the normalised and difference plots. The Precision Melt Software v. 1.2 (BioRad) then assigned each sample to a cluster (Clusters 1–3) using default settings. Single dog sample was assigned to *A. reconditum* (*light green*; note the ambiguous melting profile in differential melting graph **b**), however DNA sequencing matched *D. immitis* 12S rRNA gene reference sequence (FN391554)

In total, 36 FVS-CN samples (36/39) returned < 40 C_t_-value (Additional file 1: Table S1). The calculated microfilarial concentration ranged from 1 (FVS-CN#24) to 44,957 (FVS-CN#36) microfilariae/ml (Table 1). Three distinct clusters were produced with the HRM analysis of 12S rRNA gene assay performed on 35 positive samples that returned C_t_-values and positive control *Dirofilaria immitis* and *Acanthocheilonema reconditum* (Fig. 2, Additional file 1: Table S1). A single sample FVS-CN#24 was excluded from HRM analysis, because of calculated low copy number (5) and non-reliable melt profile. Cluster 1 included *D. immitis* positive control (high copy numbers) and the majority of dog blood samples (*n* = 30; 3–44,957 microfilariae/ml). Cluster 2 included *D. immitis* positive control (low copy numbers) and 4 dog blood samples (FVS-CN#10, #27, #31, #32; 15–177 microfilariae/ml). The remaining dog sample FVS-CN#3 clustered within Cluster 3 of *A. reconditum* controls, but with a variable HRM profile due to late amplification (Ct-value = 36.29) (Fig. 2; Additional file 1: Table S1). To confirm the species identification, 33 PCR products were submitted for DNA sequencing. Only 17/33 (51 %) samples yielded DNA sequencing data denoting *D. immitis* and *A. reconditum*. All DNA sequence data were identical to *D. immitis* 12S rRNA gene reference sequence (FN391554), including the suspected sample FVS-CN#3 that clustered within Cluster 3 of *A. reconditum* controls (Table 1, Additional file 1: Table S1).

### Presence of *Dirofilaria immitis* using diagnostic real-time PCR and DNA sequencing

In total 36 FVS-CN samples (36/39) were considered *D. immitis*-positive returning < 37 C_t_-value (Additional file 1: Table S1). The calculated microfilarial concentration ranged from 7 (FVS-CN#1) to 60,526 (FVS-CN#11) microfilariae/ml (Table 1). One sample (FVS-CN#14) was considered suspect for *D. immitis* with C_t_-value of 39.93 (0.035 microfilariae/ml); a repeated run yielded no C_t_-value. All positive (*n* = 36) samples yielded DNA sequences matching *D. immitis cox*1 gene (Table 1, Additional file 1: Table S1). All but sample FVS-CN#29 had DNA sequences 100 % identical with *D. immitis cox*1 gene reference sequence (NC_005305). FVS-CN#29 included unambiguous AAT > AGT (Asn > Ser) single nucleotide non-synonymous polymorphism at position 539 according to the reference gene.

FVS-HW#1, FVS-HW#1 and FVS-FIJI#1 returned positive result of *D. immitis* (C_t_-values: 25.76, 29.82 and 35.47, respectively), DNA sequences were 100 % identical with *D. immitis cox*1 gene reference sequence (NC_005305).

### Wildtype form of *Dirofilaria immitis* P-glycoprotein locus in Australia

Wild-type genotype AA-GG of the P-glycoprotein locus of *D. immitis* (P-gp) sequence has been obtained for three blood samples (rigorous prevention group: FVS-CN#11, FVS-CN#34 and inconsistent prevention group: FVS-CN#37). Other samples did not yield sufficient P-gp PCR product for DNA sequencing. Whole genome amplification prior PCR amplification using P-gp primers yielded DNA sequence with top BLAST hits from the dog genome.

## Discussion

It is important for the control of canine heartworm that preventatives remain efficacious, and compliance with treatments and heartworm antigen testing is practised. In this study, a large proportion of the dogs with microfilariaemia were reportedly on rigorous monthly heartworm prevention with either milbemycin oxime, ivermectin or selamectin. All monthly heartworm preventatives undergo rigorous experimental testing prior to their registration [31]. It is expected that such preventatives are demonstrated to have 100 % efficacy [31].

Two important caveats need to be taken into account when considering rational prevention and compliance: (i) residual activity and (ii) age of *D. immitis* present in the dog. For (i), the residual activity of milbemycin oxime, ivermectin or selamectin lasts only several days, therefore it is expected that monthly doses remove all larval stages that accumulated over the past 30 days, implying that all *D. immitis* need to be less than 30 days old. Subsequently, the efficacy for *D. immitis,* of the various MLs depends on the age of the parasite (ii). The efficacy is 100 % for 30 day-old *D. immitis* based on registration for products with milbemycin oxime, ivermectin or selamectin. A single dose of ivermectin or selamectin applied 60 days after the infestation remains 100 % efficacious but decreases to 95 % for milbemycin oxime (see Table 1 in [32, 33]). In other words, compliance with monthly administration has to be strict with milbemycin oxime, because comparative studies have shown that milbemycin oxime is not as effective for ≥ 30 day-old *D. immitis* in dogs when compared with ivermectin [8, 33, 34]. Therefore, if a monthly treatment with milbemycin is missed, a small proportion of *D. immitis* may be able to develop to adults and produce microfilaria.

An incomplete compliance is a real possibility in our cohort, because a large portion of *D. immitis*-positive dogs was on milbemycin oxime. Inadvertently missing three consecutive monthly doses means potentially four month-old *D. immitis* exist before the next 12 doses are applied; in such circumstances, McCall et al. [34] demonstrated only 41 % efficacy of milbemycin oxime against *D. immitis*. Alternatively, it can be a reflection of larger market share of products with milbemycin oxime for heartworm prevention over other MLs in the locality. To document any loss of efficacy to MLs, a full history of prevention and annual antigen test would be required [35]. Therefore, a reduced efficacy of MLs in these Australian cases must be treated as ‘remotely possible’ due to incomplete information [21].

The putative ‘rigorous’ canine heartworm prevention should have halted development of *D. immitis* adults and abrogated any microfilariaemia. Loss of efficacy for milbemycin oxime, ivermectin or selamectin would be a major concern for veterinarians, pet owners and suppliers in Australia. The current study documents the presence of a focus of heartworm prevalence, but much more complete histories and testing regimes are needed to indicate whether resistance is present or developing in the region. In future suspected cases in Australia, a ‘microfilaria suppression test’ should be considered [23]. For example a microfilaricide, either ivermectin (50–200 mg/kg) or milbemycin oxime (500–1,000 mg/kg), should be applied to dogs to eliminate microfilaria. In such a microfilaria suppression test, loss of efficacy would be demonstrated, if > 75 % of microfilariae remain one week after administration of the microfilaricide [23]. Currently, it is not known whether microfilaria suppression tests have been considered for these microfilaria-positive dogs.

Only the wild-type P-gp genotype of *D. immitis* was detected in Australian samples. In this study we did not document the GG-GG P-gp genotype of *D. immitis* associated with ivermectin-resistance [7, 22, 23]. Despite the published association, any direct causal effect of the GG-GG genotype has not been demonstrated [7, 22, 23]. Our P-gp amplification varied from the original approach [22, 23]. Firstly, only stored frozen blood was available and therefore we could not assess individual microfilaria. Secondly, published P-gp amplification used full genome amplification using Repli-g® (Qiagen), while we employed GenomiPhi V2 DNA Amplification Kit (GE Healthcare). Both kits use multiple displacement amplification, but have been shown to give different yields of DNA and variable genome amplification coverage [36]. It is plausible that the use of GenomiPhi to amplify DNA caused miss-priming of the P-gp oligonucleotides and subsequent PCR amplification of non-specific products. Further investigations into the genetic identity of Australian *D. immitis* isolates are potentially valuable for comparative purposes with those from North America and Europe.

## Conclusion

For patent microfilariaemic dogs, administration of heartworm preventatives without treatment with melarsomine dihydrochloride to eliminate adult worms has the potential to select a population of *D. immitis* resistant to heartworm preventatives [31]. In Australia, heartworm prevention is ubiquitous and annual testing or testing prior yearly ML administration is only rarely undertaken, because heartworm disease is now rare. Therefore, this situation may allow for the detection of asymptomatic microfilariaemic dogs that are on monthly prevention. Most of the monthly doses will rapidly remove microfilaria, however the non-lethal effect on the adult *D. immitis* female may result in the reappearance of microfilariae before the next monthly dose is administered and especially if further doses are missed. These microfilariaemic dogs represent local reservoirs, increasing the likelihood of transmission to dogs that missed more than one monthly heartworm prevention. This scenario is plausible for the present cohort of dogs which were domiciled within a small area in coastal Queensland, Australia. Currently, no information is available about the extent of this phenomenon in Australia. These findings demonstrate that heartworm is endemic in Australia. Strict prevention according to the label of the preventative and annual heartworm test should be practiced across Australia, despite most areas are currently considered of low prevalence. The low endemic status of heartworm status in Australia is currently a limiting factor for the development of a transmission model for heartworm.

## Additional file

Additional file 1: Table S1. Summary of the *Dirofilaria immitis* diagnostics. (XLSX 16 kb)

## Abbreviations

ELISA: Enzyme-linked immunosorbent assay
HRM: High-resolution melt
ML: Macrocyclic lactone
P-gp: P-glycoprotein
SNP: Single nucleotide polymorphism
WGA: Whole genome amplification
