# Correction to: Mosquito-borne heartworm *Dirofilaria immitis* in dogs from Australia

**DOI:** 10.1186/s13071-019-3338-6

**Published:** 2019-02-19

**Authors:** Chloe Nguyen, Wei Ling Koh, Andrea Casteriano, Niek Beijerink, Christopher Godfrey, Graeme Brown, David Emery, Jan Šlapeta

**Affiliations:** 1 Faculty of Veterinary Science, The University of Sydney, McMaster Building B14, Sydney, NSW 2006 Australia; 2 University Veterinary Teaching Hospital, Sydney (UVTHS), Faculty of Veterinary Science, The University of Sydney, Evelyn Williams Building B10, Sydney, NSW 2006 Australia; 3 School of Life and Environmental Sciences, Faculty of Veterinary Science, The University of Sydney, McMaster Building B14, Sydney, NSW 2006 Australia

**Correction to: Parasites & Vectors (2016) 9:535**

**DOI: 10.1186/s13071-016-1821-x**

Since publication of the original version of this article [1], it has been flagged that unfortunately there is an error in dosage units in the **Discussion** section, in the sentence “For example a microfilaricide, either ivermectin (50–200 mg/kg) or milbemycin oxime (500–1,000 mg/kg)”.

Please be advised **that the mg units in the sentence should instead be μg**; that is, “For example a microfilaricide, either ivermectin (50–200 **μg/**kg) or milbemycin oxime (500–1,000 **μg**/kg)”.

The authors apologize for any inconvenience caused.
